# Standardized nomenclature and open science in *Human Genomics*

**DOI:** 10.1186/s40246-021-00312-9

**Published:** 2021-02-22

**Authors:** Vasilis Vasiliou, Kirill Veselkov, Elspeth Bruford, Juergen K. V. Reichardt

**Affiliations:** 1 Department of Environmental Health Sciences, Yale School of Public Health, New Haven, CT 06510 USA; 2 Department of Surgery and Cancer, Imperial College, London, SW7 2AZ UK; 3 HUGO Gene Nomenclature Committee (HGNC), EMBL-EBI, Wellcome Genome Campus, Hinxton, Cambridge, CB10 1SD UK; 4 Department of Haematology, University of Cambridge, Cambridge, CB2 0XY UK; 5 Australian Institute of Tropical Health and Medicine, James Cook University, Smithfield, Queensland 4878 Australia

Two recent papers have highlighted the vital importance of using standardized nomenclature in reporting data, especially when this is of clinical relevance. Higgins et al. [1] have drawn attention to the crucial matter of the appropriate nomenclature of DNA variants in scientific publications. It is critical that DNA variants can be identified unambiguously. And Fujiyoshi et al. [2] have called for gene products to be referenced using the approved gene symbol for the encoding gene, along with an appropriate database ID (HGNC ID, with UniProt ID where required, see Table 1 for resources for vertebrate genes). Confusion can impede data sharing and scientific progress, as well as potentially result in patient harm.

**Table 1:** Resources for standardized identifiers

*Type*	*Name*	*URL*	*Comments*
Gene symbols	HGNC (HUGO Gene Nomenclature Committee)	www.genenames.org	Human gene symbols and IDs
Gene symbols	MGI (Mouse Genome Informatics)	www.informatics.jax.org	Mouse gene symbols and IDs
Gene symbols	RGD (Rat Genome Database)	https://rgd.mcw.edu	Rat gene symbols and IDs
Gene symbols	AgBase	https://agbase.arizona.edu	Chicken gene symbols and IDs
Gene symbols	VGNC (Vertebrate Gene Nomenclature Committee)	vertebrate.genenames.org	Gene symbols and IDs for selected vertebrate species
Gene symbols	Xenbase	www.xenbase.org	Xenopus gene symbols and IDs
Gene symbols	Zfin	www.zfin.org	Zebrafish gene symbols and IDs
Protein identifiers	UniProt	www.uniprot.org	Protein IDs for multiple species

*Human Genomics* has always required the use of standardized gene symbols [3], and we now ask all authors, editors, reviewers, etc. to utilize the correct and verified nomenclature additionally for gene products and DNA variants in all submissions to this journal. Adherence to this policy will ensure full understanding by all readers and reproducibility of findings involving genes, gene products, and DNA variants. The usage of historic nomenclature in addition to this policy may be helpful in some fields to assist certain readers. We note that other journals have already taken a keen interest in these matters [4].

We also note here in the broader context that *Human Genomics* strongly encourages the sharing of data to facilitate open science (https://en.wikipedia.org/wiki/Open_science), reproducibility, and full understanding of scientific advances. Therefore, depositing all relevant omic information in general, such as genomic, epigenomic, transcriptomic, metabolomic, and proteomic data, would be of great value to the scientific community. For example, the open-access MetaboLights repository of raw experimental metabolomic data and associated metadata has been recently re-designed to facilitate the growing demand for reproducibility and integration with other “omics” [5]. The recently engineered auto-deconvolution MSHub/GNPS platform has further enabled the community to store, process, share, annotate, compare, quantify reproducibility, and perform molecular networking of mass spectrometry metabolomic data in the context of multi-omics studies [6]. Accordingly, we strongly encourage depositing all relevant omic data relating to publications in our journal to aid advancement and reproducibility in science (Table 2).

**Table 2:** Examples of relevant omic databases

*Type*	*Name*	*URL*	*Comments*
DNA Variants	dbSNP	https://www.ncbi.nlm.nih.gov/snp/	NCBI SNP (single nucleotide polymorphism) database
	ClinVar	https://www.ncbi.nlm.nih.gov/clinvar/	NCBI database on genomic variation and clinical phenotypes
	LOVD (Leiden Open Variation Database)	https://www.lovd.nl	Open database for genetic variants
Proteomic	Protein	https://www.ncbi.nlm.nih.gov/protein/	NCBI protein databases
Proteomic	Human Variants Database	https://www.iitm.ac.in/bioinfo/huvarbase/	Human protein variants
Metabolomic	Metabolights	https://www.ebi.ac.uk/metabolights/	Open database of metabolomic experiments, derived metabolites, their structures and biological roles
Metabolomic	GNPS	https://gnps.ucsd.edu	Mass spectrometry driven small molecule analysis
